# DNS/DANE Collision-Based Distributed and Dynamic Authentication for Microservices in IoT †

**DOI:** 10.3390/s19153292

**Published:** 2019-07-26

**Authors:** Daniel Díaz-Sánchez, Andrés Marín-Lopez, Florina Almenárez Mendoza, Patricia Arias Cabarcos

**Affiliations:** 1 University Carlos III de Madrid, 28911 Leganés, Spain; 2 University of Mannheim, 68161 Mannheim, Germany

**Keywords:** IoT, microservices, DNSSEC, DANE, chameleon signatures

## Abstract

IoT devices provide real-time data to a rich ecosystem of services and applications. The volume of data and the involved subscribe/notify signaling will likely become a challenge also for access and core networks. To alleviate the core of the network, other technologies like fog computing can be used. On the security side, designers of IoT low-cost devices and applications often reuse old versions of development frameworks and software components that contain vulnerabilities. Many server applications today are designed using microservice architectures where components are easier to update. Thus, IoT can benefit from deploying microservices in the fog as it offers the required flexibility for the main players of ubiquitous computing: nomadic users. In such deployments, IoT devices need the dynamic instantiation of microservices. IoT microservices require certificates so they can be accessed securely. Thus, every microservice instance may require a newly-created domain name and a certificate. The DNS-based Authentication of Named Entities (DANE) extension to Domain Name System Security Extensions (DNSSEC) allows linking a certificate to a given domain name. Thus, the combination of DNSSEC and DANE provides microservices’ clients with secure information regarding the domain name, IP address, and server certificate of a given microservice. However, IoT microservices may be short-lived since devices can move from one local fog to another, forcing DNSSEC servers to sign zones whenever new changes occur. Considering DNSSEC and DANE were designed to cope with static services, coping with IoT dynamic microservice instantiation can throttle the scalability in the fog. To overcome this limitation, this article proposes a solution that modifies the DNSSEC/DANE signature mechanism using chameleon signatures and defining a new soft delegation scheme. Chameleon signatures are signatures computed over a chameleon hash, which have a property: a secret trapdoor function can be used to compute collisions to the hash. Since the hash is maintained, the signature does not have to be computed again. In the soft delegation schema, DNS servers obtain a trapdoor that allows performing changes in a constrained zone without affecting normal DNS operation. In this way, a server can receive this soft delegation and modify the DNS zone to cope with frequent changes such as microservice dynamic instantiation. Changes in the soft delegated zone are much faster and do not require the intervention of the DNS primary servers of the zone.

## 1. Introduction

Internet of Things (IoT) devices are equipped with multiple sensors that gather data from the user or the surrounding physical world. From health devices to sports wrist-worn devices and domestic appliances, these devices provide real-time data to a rich ecosystem of services and applications, which can personalize the user environment as expected in the ubiquitous computing scenarios. The volume of data and the involved subscribe/notify or polling signaling will likely become a challenge for both access and core networks. Many designers have identified cloud computing, edge computing or fog computing as a candidate to alleviate the core of the network from this background traffic. Despite that there are subtle differences, both concepts address moving data closer to the user and perform some processing near the client requiring the instantiation of new services. We address the protection of those services, so due to that, from now on, we will refer only to the fog. This hypothesis is satisfied since the majority of the IoT traffic is expected to be exchanged in the vicinity of the devices, though some interactions will require traversing core networks to persist consolidated data.

IoT devices have a second characteristic: the embedded software and firmware are challenging for users to update. According to the January 2015 Federal Trade Commission report [1] on IoT device security, authentication APIs and system updates are the biggest problems of the IoT market. The reduced cost of many of IoT devices makes them prone to present security design flaws, due to the popularization and reuse of mature, but outdated hardware, development platforms, and software development kits. This is where the microservice modular architecture offers an immediate benefit: updating components is easier than updating the whole system (firmware). Using microservices, an application can be structured into a set of loosely-coupled services that implement different functionalities, favoring the operation and management such as the easy replacement or update of a microservice. Therefore, an application can be divided into a set of small dependent modular components that can be separately instantiated and communicate with other components by means of a communication network. Thus, microservices require strong security support for fog computing, to rely on nodes in the boundary of the network for secure data collection and processing.

It is necessary to consider that those microservices in IoT may change their location frequently since IoT involves very heterogeneous devices as personal and mobile devices and even vehicles, so near fog computing infrastructures will be used in an opportunistic way as they are discovered. In the process of instantiation, an IoT application will declare its required set of microservices, and the fog will reply with a best-effort offer to meet such requirements. To accomplish that task, these infrastructures use a dedicated component called the orchestrator. In the negotiation, the orchestrator specifies in its offer the hosting and execution of the declared services under a given quality, the address and name assignment, and may provide adequate credentials to access services securely. Thus, among the properties of a service in the fog, we can identify the following: Identification (IP or domain name resolvable within the fog); reachability (fog or even Internet); and the transparent migration, replication, or load balance of the service instance.

DNSSEC allows creating and signing the new DNS records required by the deployment negotiated by the orchestrator, so that the microservices can securely resolve and connect to other microservices. Security is obtained at the price of a high load: DNS servers have to sign every change in the zone. We propose a mechanism to minimize such load. We propose a new DNS soft delegation scheme using chameleon signatures, which removes the necessity of signing the changed DNS records. Chameleon signatures are signatures computed over a chameleon hash, which have the property: a secret trapdoor function, known only to the server that receives the soft delegation, can be used to compute collisions with the hash. Since the hash is maintained, the signature does not have to be computed again. A server is soft delegated in a zone by means of a signed record pattern, the chameleon hash of the record, and the secret trapdoor function. The server can perform changes respecting the pattern and using the secret trapdoor to compute a collision of the hash of the modified record, so that the original signature holds. We claim that since the load of computing the collision is much less than computing the signature, this soft delegation is an excellent candidate to enable IoT dynamic microservices’ deployment in a scalable way.

The structure of the article is as follows. The article reviews how microservices’ interactions can be protected by means of TLS in Section 2, discussing the network context in Section 2.1, the relation with the fog in Section 2.2, and the IoT protocols in Section 2.3. In Section 3, the article reviews certificate pinning as the current solution to enhance PKI security focusing on DNSSEC-based techniques, which will be profoundly discussed in Section 4. That section reviews the Domain Name System Security Extensions (DNSSEC) and DNS-based Authentication of Named Entities (DANE) for integrating PKI support in DNS. It also shows that DNSSEC and DANE are not ready to cope with the dynamicity of IoT and M2M, so a change in the signature process of DNSSEC using chameleon signatures, discussed in Section 5, is proposed in Section 6 to enable secure communication with standard protocols for dynamically-instantiated microservices. Results and conclusions are presented in Section 7 and Section 8, respectively.

## 2. TLS and Microservices in IoT/M2M

### 2.1. IoT, M2M, and Related Network Technology

There is no consensus around what is the Internet of Things and for what it can be used. However, the Internet of Things (IoT) [2] can be seen as a very general concept of connected devices that encompasses related concepts such as Machine-to-Machine (M2M) [3], smart cities [4] and crowd sensing [5], among others.

These concepts are interchangeable [6] according to the scope of the article that mentions it. Smart cities, which lacks a clear definition [7], aim at transforming the environments in which life and work are developed focusing on a concrete region, usually a city. To accomplish that, smart cities incorporate a wide variety of digital, electronic, and data processing technology into a management or governance system [8]. Crowd sensing, which has been defined in several cases as a human–machine data fusion [9], pursues resolving problems or concrete objectives in a distributed fashion, involving humans, their devices, and other elements [5].

IoT is a very general concept that considers the entire data flow from the device to the application domain, and thus, it is extensively used in the aforementioned related concepts. IoT covers the entire process from the information extraction on the device (or thing), its processing and/or fusion, including the presentation of data to other entities in order to favor an efficient use of infrastructures [10], to improve electronic participation for local governance, and to learn, adapt, and innovate with the surrounding ecosystem effectively [11]. Moreover, IoT requires scalability, due to the potential number of devices and the two-fold interactions: thing–thing and human–thing. IoT has to use enabling technologies in a cost-effective way [12]. This cost effectiveness affects every element of the data flow, from the design of connected devices to the data processing system. Thus, efficient data processing systems are of paramount importance in IoT, making elastic computing systems [13] essential when transforming unprecedented volumes of data [14,15] into consolidated information.

Furthermore, the diversity of applications in IoT obliges networks to consider heterogeneous traffic patterns, so flexible network architectures are also a pressing need. This flexibility should exist at both the network core and endpoints to transport data from devices and intermediary elements to the application domain. To accomplish that, these infrastructures should be interoperable, trusted, secure, and privacy preserving.

Several standardization bodies have defined M2M [16] architectures to bring interoperability. M2M can be considered an evolution of Supervisory Control and Data Acquisition (SCADA) systems, but also encompasses concepts from cellular and Wireless Sensor Networks (WSN). Like traditional WSN that usually serve a single purpose or a single organization, M2M networks have been designed to improve interoperability among nodes and networks, so the infrastructure can be shared and used for different purposes and applications and by different organizations. Due to that, M2M networks are expected to become important components in IoT since they can handle a huge variety of application traffic and interoperate among them.

The amount of IoT applications is expected to be so high that it will affect network performance. It should be considered that besides traditional Internet services in which traffic flows mainly from the network to end devices, IoT traffic will flow mainly the other way around. Moreover, network elements should accommodate urgent, burst, and regular traffic patterns from millions of new devices without affecting traditional services. Precisely as a way to optimize traffic, several works proposed the use of proxies and intermediate elements and other complex network services to perform traffic shaping, storing, and forwarding and interest routing to prevent service interruption [17].

Software-Defined Networking (SDN) [18] can be useful to manage distributed networks in real time. In general, SDN allows managing traffic in an effective way by separating the control plane from the transmission plane by managing different flows, but is limited to a single network or a single management point. However, in IoT/M2M scenarios, it may be necessary to redirect traffic to data sinks, the management of which is beyond the administration scope of the provider where that traffic originated. Moreover, flow redirection to intermediate storage or processing elements, as the fog or edge, may happen dynamically to overcome network congestion, whereas fulfilling the IoT application demands.

Despite these intermediate elements and management technologies bringing an additional degree of freedom, it is necessary to consider that data managed by IoT/M2M networks may contain personal data either generated by personal devices, as may happen in scenarios with personal IoT devices and crowd sensing applications, or in many scenarios in which devices produce data from the observation of the physical world, as happens in smart cities. The resulting collected data may raise privacy and confidentiality concerns requiring adequate security mechanisms for both data consolidation and interaction among devices and microservices.

### 2.2. Fog Computing and Microservices

Cloud computing (or the cloud) infrastructure, which is located in the operators’ backends, is gradually migrating to the edge of the network and beyond, to cope with an increasing number of client devices. As has also happened with other concepts, as web services, smart cities, and big data, in the beginning, this kind of concept transformation may receive different names. However, interesting literature can be found regarding how these topics are classified and what can be expected from them [19,20]. Like other concepts, fog computing (or the fog) is not restricted to a single technological area, but focuses on general aspects such as scalability and interworking. The problem of fog computing is that it embraces several technological trends, thus making it complex to find a clear definition [20].

**Figure 1:**
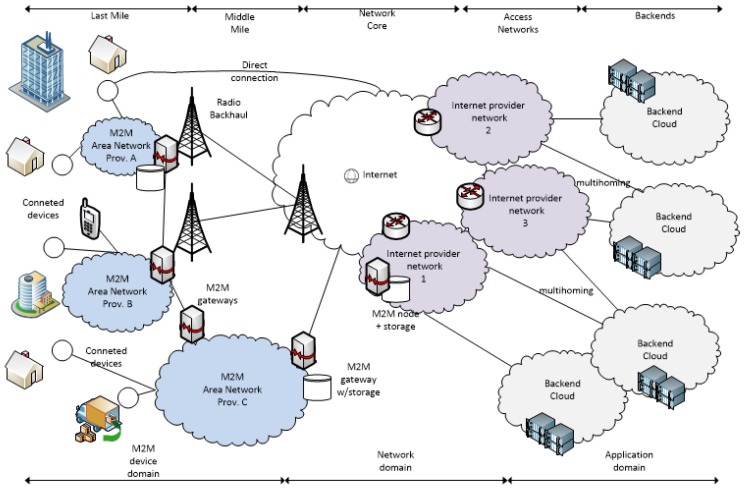
IoT/M2M network infrastructure in which different devices interconnect with network nodes near them and with the backend [24].

Fog computing is presented in many cases just as an evolution of the cloud computing model [21]. This definition of the fog as an evolution of the traditional cloud, that moves to the network edge, to provide services to millions of devices, brings us closer to the concept of the fog. The most distinctive characteristics of the fog versus the cloud are the geographical proximity to end users and end devices, its distribution in wide geographic areas, and its support for mobility. Considering the increasing ecosystem of IoT/M2M devices, the most interesting characteristic of the fog is the proximity to the end devices. This increasing number of devices, conservatively estimated to be 50 billion devices [14], can be divided in two groups, which are personal devices and sensor/actuator devices, all of them spread in a very wide area. Those devices are components of IoT and its related concepts such as smart cities, smart metering [22], wearable computing [23], crowd sensing, and M2M (see Figure 1).

Network elements in any IoT/M2M computing infrastructure, as fog computing nodes, should be able to give support to constrained devices and support narrowband communications. Bluetooth, which was the first affordable Personal Area Network (PAN), supports very limited use cases, and it is not a reasonable candidate for the IoT/M2M network due to its consumption; however, it is pervasively supported. Other PAN technologies, such as ZigBee [25], were specifically designed for low bandwidth networks (Low-Rate Personal Area Networks (LR-WPAN)), supporting more use cases than Bluetooth, but with bandwidth limitations. Bluetooth Low Energy (BLE), which was designed keeping the data transfer rates of Bluetooth, offers other operation modes that fit better in an IoT/M2M ecosystem. Other recent standards [26], such as 802.15.4e, 802.15.4f, and 802.14.4g, and de facto technologies, such as Z-Wave, DASH7, or wireless M-bus, some of them with scarce adoption [27,28], suggest network nodes in fog computing should support a wide spectrum of communication technologies to favor cooperation among devices and between devices and the application domain.

As has been reasoned before, other devices to collect, store, aggregate, or filter device data and eventually deliver added value data to the application domain should also exist, i.e., gateways. As discussed in Verma et al. [3], several differentiated traffic patterns in IoT/M2M networks allow network operators to give priority to several operations over others to reduce congestion and latency at the network core. There are profound discussions concerning traffic patterns [3,29,30,31], but for the purpose of introducing the fog, it is possible to distinguish two groups. The fist corresponds to applications demanding immediate delivery or having any time constraint. Some examples of applications that require immediate delivery can be emergency, critical system monitoring, and alert systems, among others. Others, such as surveillance, multimedia distribution, or real-time video communication, are examples of time-constrained services in which latency, bandwidth, or other parameters should be kept stable. The second group corresponds to adaptive traffic applications, which are those that do not impose restrictions in latency or bandwidth, so they are tolerant to variations in these parameters. Some examples are smart grid measures, environmental monitoring, vending, and many others excluding alarms, or urgent notifications generated by these systems. Thus, there are many generated data that deserve to be processed, but not immediately, so they can be conveniently stored at intermediate nodes or partially processed according to the application without traversing the network core, and fog computing is a key element for that.

Managing this increasing number of end and intermediary devices goes beyond current network management limits. Function Virtualization (NFV) [32] is among the most outstanding efforts focusing on solving this problem. It allows operators to provide reliable complex communication systems on demand. NFV provides a boilerplate architecture where complex services, such as, for instance, firewalls, WAN accelerators, and switches, but also other user services, such as a database, can be instantiated [33]. The combination of SDN and NFV enables the creation of virtual networks within a local cloud [34]. Furthermore, Long-Term Evolution [35], Enhanced Packet Core (EPC), and 5G [36], which plans providing a universal coverage area, set out moving computing power near the user. Edge computing is like the concept of fog computing, but closer to the operator, and sometimes interchangeable. In fact, there are many fog services, thought to be instantiated very close to the user, that could be also instantiated in edge computing. Finally, fog nodes can be found far from the network, but communicated with it by means of radio backhauls. Sometimes, these isolated fog nodes have been described as “mist” [37].

Reusing hardware platforms, drivers, and even programming frameworks to build IoT/M2M devices worked out well for a rapid growth of the IoT market, but also caused worrying security problems since vulnerabilities found in a device can be quickly exploited in a big population of devices as they share either hardware of software. Moreover, there are significant disparities in the support quality and update frequency of devices. Whereas expensive connected devices are frequently updated to fix vulnerabilities, many other low-cost devices are scarcely updated. This is one of the reasons why microservices and the fog can alleviate this problem, as they not only provide computation resources, but also low latency due to their proximity to end devices.

Microservices [38] organize an application as a collection of loosely-coupled services, each of them implementing part of the application. Microservices’ modular architecture allows instantiating components separately, or in different places, also allowing for continuous delivery [39] since different components can be updated independently. Thus, it eases management and development operations [40] and favors agile development [41]. Microservices can also help to reduce the part of the application implemented in the devices by the instantiation of other functionalities in fog or edge computing on demand [42]. Since microservices are components that can be independently developed and published in repositories, devices can have access to the most recent versions of their components as they can be fetched at execution time. In this way, security problems originated from a lack of updates of IoT devices and applications may be minimized and attack surfaces reduced.

### 2.3. Protocols in IoT/M2M

Microservices can be used in IoT/M2M scenarios to address several security problems. However, other security and management requirements can be derived from the protocols used to transport data. IoT/M2M devices will have constant communication with cloud computing, edge computing, or fog computing infrastructures. This communication may range from communications between components of distributed applications (several microservices) or just communications for persisting or transporting data to the cloud. The biggest amount of traffic will be concentrated in devices’ surroundings arising from cooperation among devices, and between devices and fog/edge/cloud computing backends. However, despite being less numerous, interactions between devices and backends for data consolidation will also be frequent.

It is also worth considering that the traffic generated among devices and services may contain personal or critical information and should be adequately protected. Infrastructures should at least provide service authentication and confidentiality. It is also critical to provide support for micro-service dynamic authentication, since many of these services will be instantiated on demand.

IoT/M2M protocols have been designed to solve application domain problems rather than security problems. This does not mean these protocols lack security support, but they rely, typically, on TLS [43], or its Datagram version (DTLS) [44], for end-to-end authentication and protection. In fact, HTTP, CoAP, Quick UDP Internet Connections (QUIC), among other applicable protocols in the context of IoT/M2M [3], use TLS or DTLS for confidentiality and authentication [45].

The reason is that, despite there being many protocols enabling authentication in distributed environments, the only protocol that has been pervasively used for authenticating end-to-end communications is TLS [43]. IPSECcan provide the same services as TLS or complement it, but in practice, it is relegated to the establishment of tunnels and virtual private networks. Regarding IoT communications, considering many of them can be opportunistic, TLS is an excellent tool to create secure channels. It requires devices to process public key certificates, but is quite versatile and has been the grounds for many IoT protocol proposals.

TLS was originally designed to work on top of a TCP since it needed an underlying connection-oriented protocol. However, there are versions of TLS working over UDP [44] and SCTP. Moreover, encryption mechanisms can be negotiated at the beginning of a TLS session, and other functionalities can be added by means of the TLS extension mechanism [46,47,48].

Despite TLS or DTLS being known to be very competent security protocols, several aspects of their design may complicate their use with IoT/M2M applications as they were designed for static services, such as web or email services. As has been discussed, IoT/M2M transport protocols require TLS to authenticate services that are usually identified with a domain name. Basically, a given domain name is authenticated by means of a public key certificate issued by a trusted authority for that domain [49]. This well-known mechanism is based on x509 certificates [50]. The problem is that x509 certificates were not designed for the concrete purpose of authenticating a domain name, but a X.500 [51,52] directory name. That will be discussed in Section 3.

Other problems of TLS are related to the authorities that issue public key certificates since any certificate authority is allowed to issue certificates for any domain name. This is a worrying problem that will be discussed also in Section 3.

Finally, PKI was not designed to authenticate ephemeral, but static services, so TLS should be complemented with other solutions in order to perform the authentication of dynamically-instantiated services in IoT/M2M, as proposed in Section 6.

## 3. PKI and Certificate Pinning

In PKI, there are Certificate Authorities (CA) that issue certificates used to protect communications among devices. A certificate contains a pair of asymmetric keys and a set of metadata identifying the protected service (by its domain name) and the authority that vouches for that certificate. Like other infrastructures, in PKI there is not a single trusted root that allows the verification of every existing certificate, but a set of authorities that satisfy legal and societal requirements for being considered as trusted roots. Those root certificates are incorporated into client software or operating systems in their trusted authorities’ repository as root CAs. Therefore, a CA can issue certificates for any domain name all over the world with or without the knowledge of the domain name owner. This would not be a problem of trust for a limited and audited CA space, but there has been an accelerated growth [53] of the CAs’ number and also CA misuses. Due to that, certificate pinning has been introduced as a tool to increase trust on the growing number of CAs and reduce the threats of fake certs issued by attackers.

As discussed before, TLS relies on PKI, so a client when establishing a TLS connection receives the server certificate, and the server returns a proof of possession of the corresponding private key, by signing the pre-master key that will be used to secure the session. In order to trust the server certificate, the client builds the certification chain, which allows verifying the path from the server certificate to the corresponding root CA certificate. The path may contain intermediate CAs and even intermediate certificates for accelerating and greater scalability of TLS connections. However, some solutions may violate privacy [54]. In other cases, compromised or improperly-managed CAs may issue certificates to DNS domains without agreement with the owners of such domains. Other cases happened in 2011, like the compromise of the Malaysian Agricultural Research and Development Institute and their CA used to generate a fake Adobe Acrobat updater [55]. Furthermore, in 2011, the Diginotar CA was used to issue certificates for Gmail and Facebook [56], Comodo in 2011 [57], or TurkTrust in 2013 [58], and others [59].

Certificate pinning techniques provide clients with an alternative way of increasing their trust in a server’s certificate. Some techniques define global control infrastructures together with the client cross verification. That is the case of Certificate Transparency (CT) [60] or Sovereign Keys (SK) [61]. Others pursue to limit the issuance of certificates to domains to the authority of domain owners, like DNS certification authority authorization [62]. Trust Assertion for Certificate Keys (TACK) [63] proposes a cross-verification under the control of domain owners. HTTP Strict Transport Security and the HTTP Public Key Pinning Protocol (HPKP) [64] define HTTP headers that set strict TLS policies and allow domain owners to alert clients of security compromised certificate chains. DANE together with DNSSEC [62] offers a complementary verification of PKI certificates together with DNSSEC authentication providing different use cases.

Domain name support was introduced in PKIX [65] in the SubjectAltNames extension [66]. CAs do not need to check with the domain owner to issue a certificate for a website in the domain. Precisely that is the main reason for certificate pinning techniques.

CT, SK, and TACK require collaboration among client devices to improve detecting malicious certificates. They were designed for browsers or high-end devices since timestamps and other information have to be stored and require the device’s permanent connection for receiving and processing similar information from other devices.

Certificate pinning solutions for IoT/M2M have been profoundly revised by the authors in previous works [67]. Nevertheless, we argue that actual certificate pinning techniques do not offer the dynamicity required by IoT microservices. An extensive analysis of the challenges of PKI and TLS for very dynamic environments as IoT and M2M shows that there are very few solutions able to authenticate a constantly-changing service ecosystem. Moreover, despite that many others can be adapted to support ephemeral credentials or certificates, only DANE currently supports security islands comprising self-issued and domain-issued certificates.

DANE brings some characteristics together that are desirable for dynamic IoT environments, as it provides a defense against CA misuse, whereas it provides an alternative certificate validation not limited to CA-issued certificates. The only requirement of DANE is to make a DNS request before connecting in order to fetch DNS registries that will be used for validating certificates. However, considering that a DNS request should be made before connecting to resolve an IP address from a domain name, it is not an important problem. DANE is especially interesting for IoT since it does not require additional trusted third parties to work and also allows domain owners to manage trust, indicating what CA should be trusted for the domain, or even specifying the exact certificate a TLS server should present. Moreover, as mentioned before, it supports the use of local or domain CAs and self-signed (ephemeral) certificates to protect TLS servers.

IoT applications requiring services instantiation in the fog may receive identifiers, such as IP addresses and domain names. Domain names are preferred since they are easier to reuse, re-instantiate, move, replicate, and load balance across the nodes of the fog. This is a consequence of the fog DNS controlling the IP assignment to a domain name in the fog zone, and thus easier control of balancing or service migration. DNS provides the required dynamicity of these cases, and it is complemented by DANE. The next sections show how DANE can bring security for accessing the dynamically-deployed nodes with the help of chameleon signatures.

## 4. DNSSEC and DANE

**Figure 2:**
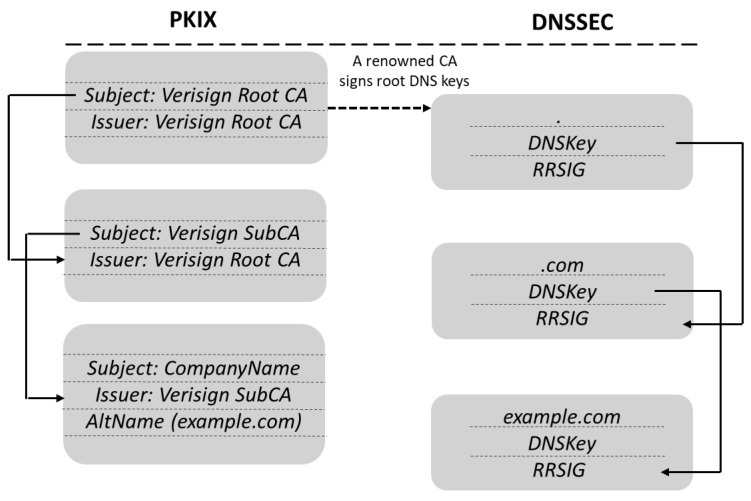
Comparison of verification chains in DNSSEC and PKIX. Image from [70]. CA, Certificate Authority.

DNSSEC [68,69] introduces a linkage of PKI credentials to domain names. DNSSEC offers DNS resolvers authentication of DNS data, which can be verified following a verification path up to the root DNS servers, authentication of the non-existence of a domain name, and the integrity of DNS data. DNS Resource Records (RRs) in DNSSEC are signed and the signature included in an additional registry called the RRSIG. The Zone Signing Key (ZSK) used to sign RR is in its turn signed with the Key Signing Key (KSK), which is available at the parent zone. DNSSEC defines the Delegation Signer (DS) resource records, which contains the KSK of the child zone to allow delegating the security of delegated zones, as required to maintain DNS scalability, and also to build a certificate chain alternative to PKI up to the DNS root (the empty domain), which must be signed by some recognized authority, i.e., some of the root CAs (see Figure 2).

DANE [62] supports clients who need to TLS connect to a server End Entity (EE) in the domain zone. DANE allows associating DNS Resource Records (RR) of type TLSAto domain names indicating a credential for the server: the credential that the server must use in a TLS connection (PKIX-EE); a domain name Trust Anchor (TA) that links the CA authorized to issue certificates for the domain (PKIX-TA); a local CA entitled to issue trusted certificates for the domain (DANE-TA); and even to indicate an ad-hoc key for TLS (DANE- EE). DANE-TA and DANE-EE establish trust without PKI intervention, but from the verification of DNSSEC pointing to TLSA records.

A DNSSEC server can modify its zone to support the dynamic microservices’ deployment required by IoT, but this introduces two main problems.

First, every change, even if it is a single TLSA record, requires updating the zone signatures. This is required for other microservices and IoT devices to rebuild the certificate chain and confirm the trust in a changing microservice identification. Computing the new signatures with every minor change is a high load for the DNSSEC server; even worse, the signatures must be computed while the TLSA record is changed, to ensure the proper verification of the whole scenario.

**Figure 3:**
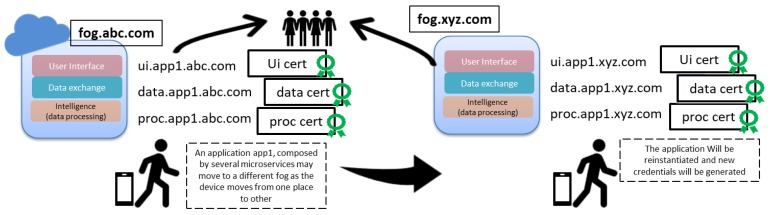
An application (app1), composed by several microservices, may move to a different fog as the device moves from one place to other requiring the re-instantiation of those microservices and the generation of new credentials.

Second, DNS servers are a critical resource of a domain. Attending resources’ frequent updates may compromise DNS service availability, especially if updates require expensive processing like cryptographic computations, which may happen when a device moves from one place to other and microservices should be re-instantiated in another fog, as shown in Figure 3. If TLSA records are to be updated frequently, this may kill the performance of the DNS server, and we do not expect fog dynamic scenario management to compromise the availability of the core DNS service.

Even more, using the core DNS to sign every small change hampers the desired distributed and best-effort management. The fog should perform a best-effort management of the microservices offloaded from the core central system management. To overcome this problem, we propose to change the signature of DNSSEC making use of chameleon signatures, which are explained in Section 5, allowing the required dynamicity without requiring so frequent updates of the DNSSEC zone.

## 5. Chameleon Signatures

Chameleon signatures [71], as other more popular signatures (ECDSA, etc.), provide the undeniable commitment of the signer with respect to a signed document that is sent to a receiver. The main difference is that chameleon signatures cannot be transferred by the receiver to a third party without the signer’s involvement. The signer is thus required to participate in checking the validity of the signature.

Chameleon signatures are proven secure under the standard model. Chameleon signatures use a special hash algorithm, the chameleon hash, which has the property of being collision resistant for the signer, but the receiver can compute collisions at will. Chameleon signatures introduce a trapdoor, which can be used to find collisions in such a way that the signature of signer S allows the receiver R to generate additional S signatures at will. Sending a chameleon signature requires R to be trusted by S. Nevertheless, S is the only entity who can prove that a fake signature is indeed a fake. However, S cannot show that a legal signature is fake.

Chameleon signatures are not transferable in general, i.e., they can only be checked by a single receiver. Nevertheless, the receiver can transform them into a transferable version [72] by publishing partially or totally the original message, since the collisions’ trapdoor is generated using R’s public key.

Let us illustrate how the chameleon signatures work using discrete logarithms, as taken from [73].

Let *R* be a receiver, *q* be a large prime, and p=kq+1, and let gbe a generator of $Z_{p}^{*}$. Choose *R* private key $SK_{R}=x\in \left(1,q-1\right)$ and the corresponding public key $PK_{R}=y=g^{x}\mathrm{mod}\;q$. Let ($PK_{S}$,$SK_{S}$) be the pair of public and private keys of the signer *S*.

The chameleon hash of a message *M* takes two random numbers *r* and *s*, and it is computed over the hash of a message and *r*, for instance $e={\mathrm{SHA}}_{256}\left(M,r\right)$. The chameleon hash is defined in Equation (1).

(1) $${\text{CHAM-HASH}}_{R}\left(M,r,s\right)=\left\{r-\left(y^{e}g^{s}\mathrm{mod}\;p\right)\mathrm{mod}\;q\right\}$$

Given the message *M* and the chameleon hash Hash, *R* asks *S* to sign the chameleon hash using $SK_{S}$. The signature $sig=SIG_{S}\left(CHAM-HASH\right)$ together with (m,r,s) can be verified using $PK_{R}=y$ and $PK_{S}$, the public keys of *R* and *S*. First, we compute $e={\mathrm{SHA}}_{256}\left(M,r\right)$, then $H2=r-(y^{e}g^{s})\mathrm{mod}\;q$. Finally, we check $VRFY_{PK_{S}}\left(sig\right)=H2$, and if this holds, the signature is valid.

When *R* wants to find a collision over a new message $M^{\prime }$, *R* finds a new random k∈(1,q−1). Next *R* computes the collision for the chameleon hash as:

(2) $$r^{\prime }={\text{CHAM-HASH}}_{R}\left(M,r,s\right)+\left(g^{k}\mathrm{mod}\;p\right)\mathrm{mod}\;q.$$

Now, *R* computes the new hash of the message as $e^{\prime }={\mathrm{SHA}}_{256}\left(M^{\prime },r^{\prime }\right)$, and $s^{\prime }=k-e^{\prime }x\mathrm{mod}\;q$. The new message can be sent together with the original signature for verification $\left(sig,M^{\prime },r^{\prime },s^{\prime }\right)$, since $r^{\prime }$ and $s^{\prime }$ are being computed to preserve the chameleon hash.

The verifier computes $e^{\prime }={\mathrm{SHA}}_{256}\left(M^{\prime },r^{\prime }\right)$, then $H2^{\prime }=r^{\prime }-\left(y^{e^{\prime }}g^{s^{\prime }}\right)\mathrm{mod}\;q$. Substituting $r^{\prime }$ from Equation (2), $H2^{\prime }={\text{CHAM-HASH}}_{R}\left(M,r,s\right)+\left(g^{k}\mathrm{mod}\;p\right)-\left(y^{e^{\prime }}g^{s^{\prime }}\right)\mathrm{mod}\;q$. Substituting the expression of the original chameleon hash and $s^{\prime }$, we get $H2^{\prime }=r-y^{e}g^{s}+g^{k}-y^{e^{\prime }}g^{k-e^{\prime }x}\mathrm{mod}\;q$, and since $y^{e^{\prime }}=g^{e^{\prime }x}$, we get that $H2^{\prime }=H2$, the same one we computed in the original verification; the signature holds.

We will follow this approach and publish the message and random numbers together with the signature to allow other parties to verify the signature.

## 6. DNS Secure and Dynamic Authentication Supporting IoT Microservices

The fog or cloud infrastructure where the IoT applications are to be deployed has one or many primary DNS (or secondaries servers) with authority for the DNS zone(s). We will refer to this (these) as the core DNS. To address the dynamicity imposed by the microservices, we propose that the core DNS instantiates a new microservice: the fog DNS. The fog DNS plays the role of a DNS forwarder, i.e., it intercepts requests for providing DANE authentication of dynamically-instantiated new microservices and authenticating existing fog and external services. This fog DNS allows the required dynamicity and prevents the core DNS (at the core of the network) from handling numerous requests from the fog since the availability of the core DNS is critical for the normal network operation.

To achieve this, we propose a soft delegation of zones to be managed by others DNS servers, but without the effective delegation of the zone. Delegated DNS servers will play the role of DNS forwarders, intercepting DNS requests to be resolved within the fog. The logical consequence is that the management will be decentralized from the core of the network, concentrating a considerable amount of traffic in the fog.

By soft delegation we refer to a schema where the delegate (the one to which the zone is soft delegated) can dynamically incorporate new DNS records into the zone on behalf of the core DNS, at the request of the microservice dynamic deployment, re-instantiation, etc. The incorporation of new records must allow for the verification of credentials, requiring records to be signed, and the corresponding signature incorporated into RRSIG records. Moreover, the whole zone has to be signed when a single new record is incorporated. We used TLSA DANE records protected with DNSSEC by the fog DNS, and that requires the fog DNS to be able to generate signatures as the core DNS, but without having access to the keys of the core DNS, neither KSK nor ZSK.

**Figure 4:**
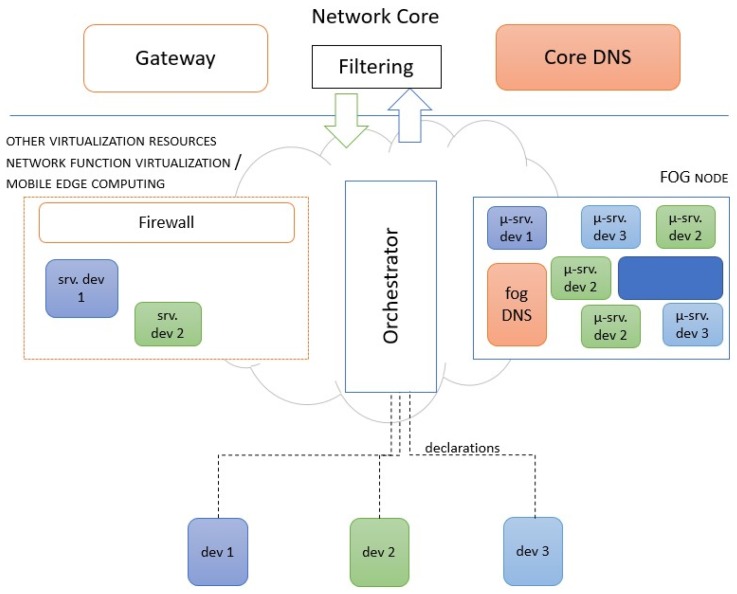
Fog DNS as a microservice in a node filtered and firewalled from the core of the network.

The fog DNS is a service that can be instantiated in an opportunistic way in a computation node for microservices, in the virtualization infrastructure, or in the fog, as illustrated in Figure 4. In that figure, services that require visibility from the Internet (out of the fog) must use the gateway. This is something that has to be specified when they are declared for instantiation.

There may be several fog DNS when several zones are soft delegated, though a fog DNS can cope with several soft delegated zones. When the load is too much, the orchestrator will ask the core DNS to instantiate a new fog DNS to load balance with the existing one(s).

### Soft Delegation Proposal

DNSSEC responses conveying one RR include the RRSIG with the signature of the RR. The resolver must use the DNSKEYRR to authenticate the RR sets. The proposed soft delegation proposes to use a pattern RRSIG pre-signed with the DNSKEY of the core DNS and delegated to the fog DNS. The signature will involve a chameleon hash so that the fog DNS can compute collisions for the dynamic modification of records.

When a new fog DNS is instantiated, the core DNS will receive the request from the orchestrator with the name and the IP assigned. The core DNS will generate the new pattern with the domain being soft-delegated and the RRSIG including the chameleon hash using the public key of the fog DNS and signing the RRSIG with the core DNS secret key.

**Figure 5:**
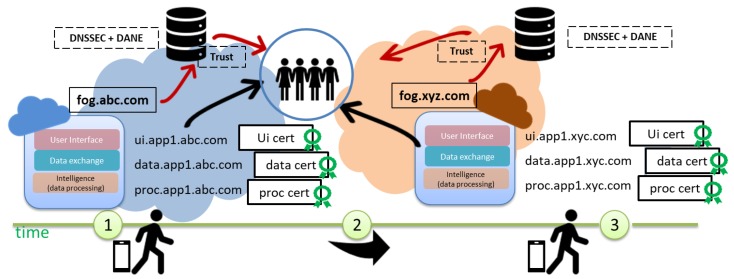
Fog DNS servers will be trusted by clients as they hold DNSSEC credentials delegated by core DNS servers. Thus, trust will be handled locally by fog DNS servers.

Microservices will experience frequent changes due to creation, migrations, and re-instantiations. The orchestrator requests those changes directly from the fog DNS. The fog DNS will compute the collision and modify the RR record accordingly, so that the RRSIG signed originally by the core DNS holds. Resolvers can thus verify the new RR, and the whole procedure is done without involving the core DNS. For instance, consider a device that that moves from fog abc.com to xyc.com, as shown in Figure 5. Microservices can be easily instantiated in a new fog, and new credentials will be provided as well.

The core DNS will be the signer *S*, as explained in Section 5. The core DNS generates a pair of public and private keys called $PK_{core}$ and $SK_{core}$. It will include a DNSKEY record with the hash of $PK_{S}$ that will be identified as the zone signing key in ZSK, which will also include a key tag that we will call $KT_{R}$ in reference to the participant (receiver or delegatee) *R*. The $KT_{R}$ tag has to be signed by the KSK of the core DNS, to extend the trust in the core DNS key to this new key.

The core DNS instantiates a fog DNS that will play the receiver role *R* as explained in Section 5 with a fresh pair of public and private keys $\left(PK_{FOG},SK_{FOG}\right)$.

The core DNS generates a pattern record that limits the delegation of the fog DNS. The pattern will reflect the subdomains that can be altered by the fog DNS. Let xyz.com be the domain of the core DNS. In Table 1 and Table 2, we show some examples of the pattern in textual format. The patterns allow the soft delegation and limit the capability of the fog DNS to alter records not delegated by the core DNS.

The core DNS sets and signs the pattern record so that the signature can be transferred to a resolver. The resolver can thus verify the core DNS signature and determine that the new data of the records filled by the fog DNS match the pattern set by the core DNS.

The record pattern format is a proposal to improve readability, but it may be replaced by other mechanisms like a Bloom filter [74], including the disallowed registers or a hash and URL to a security policy.

Table 1 shows the example fields of the pattern record limiting the fog DNS. <T> represents a positional marker of a final label, and [.L] allows optional delegation of whole subdomains without a zone delegation. MAX_TTL sets the maximum TTL to records associated with the pattern. <R> is a positional marker to indicate the type of record this pattern allows to verify. Finally, rnd is a random number selected by the core DNS. Table 2 shows particular examples of the pattern record to illustrate its usage.

The core DNS (*S*) generates the signature for the pattern record as shown in Table 3.

The fog DNS configured by the core DNS can now intercept fog incoming requests and resolve them with authenticated records as shown in Table 4.

Verification by a resolver requires the pattern record, and also ($PK_{FOG}$, *g*, *p*, *q*, *r*, *s*), that will be included as DS-type records using private OIDs, not requiring any additional protocol.

${\mathrm{PK}}_{FOG}$ does not require to be certified by a trusted CA, using DANE-TA or DANE-EE (as explained in Section 4. Often, the public key of the fog DNS will be ephemeral in highly dynamic environments where the fog DNS requires frequent re-instantiation operations.

Once the resolver has verified the ZSK ${PK}_{core}$ of the core DNS, it only has to perform several DNS requests [69] to obtain the pattern record, *r*, *s*, *g*, *p*, *q*, and ${\mathrm{PK}}_{Fog}$, from the fog DNS or using other protocols besides DNS to discover services like NetBIOS or WS-discovery, for example. The resolver is then able to verify the signature of records from the fog DNS.

Let us present an example. Let the fog DNS receive a request to add an A-type RR with value 163.117.141.197 associated with domain name srv.xyz.com. The fog DNS will create the corresponding DNS record as shown in Table 5.

Let *Q* be a resolver sending a query of type A for the domain name srv.xyz.com. The request is intercepted by the fog DNS that gets the information contained in Table 6.

**Figure 6:**
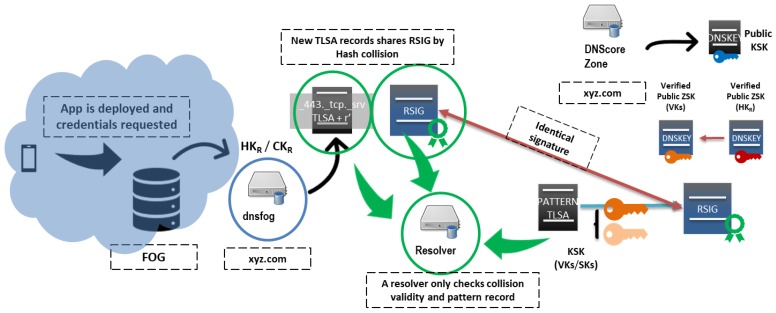
The fog DNS receives delegation from the core DNS server, so it can find hash collisions and reuse RRSIG records from core DNS servers to authenticate ephemeral TLS records. A resolver would only need to check the validity of the collision and the pattern record as it trusts the core DNS DNSSEC credentials. KSK, Key Signing Key; ZSK, Zone Signing Key.

Let *Q* obtain from the fog DNS the parameters *g*, *p*, *q*, *r*, *s*, and ${\mathrm{PK}}_{FOG}$. *Q* has verified that ZSK (${\mathrm{PK}}_{core}$ of the core DNS from the previously-obtained KSK, and it knows its key tag is 32478. The verification performed by *Q* is detailed in Table 7 and illustrated in Figure 6.

## 7. Results

To show the improvement of our proposal, we compared it with the behavior of the original DNSSEC + DANE. The soft delegation only requires the steps indicated in Table 8, i.e., computing the chameleon hash and an additional signature. The standard DNSSEC delegation requires much more computation since the records for the keys of the delegated DNS and the record for the delegation of the domain name have to be created and signed in the delegating and delegated DNS.

**Figure 7:**
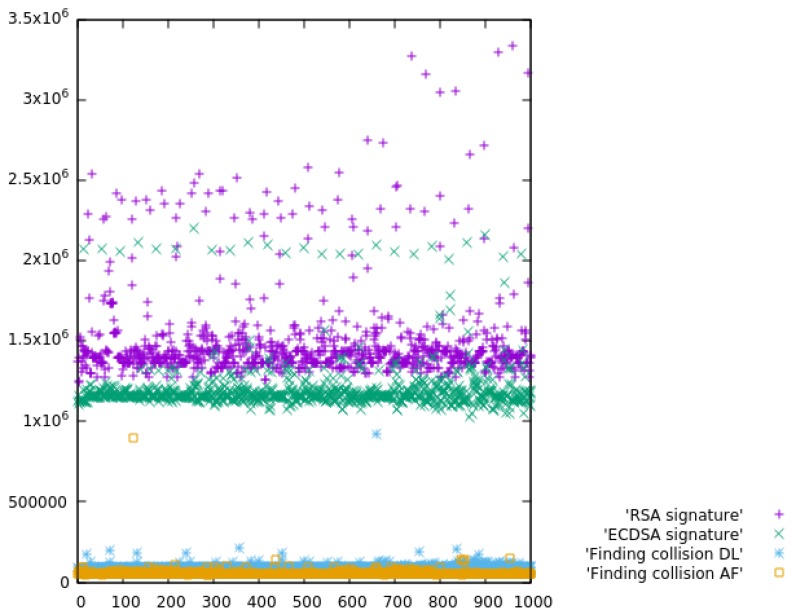
Signature computation time versus finding collision (y-axis in CPU ticks).

When a microservice is created or changed, the soft delegation only requires the steps in Table 9, since the pattern is already created and signed. The standardized DNS delegation scheme will require at least the signature (RRSIG) of the new RR, and the same if the DNS core does not delegate the domain name. Therefore, the soft delegation saves the time of performing one signature in either case and maintains the load-balancing of delegation, since the core DNS is not bothered with updates and queries about the delegated zone, preserving scalability. In the soft delegation case, instead of the time for computing a signature, it is only required to find a collision. The comparison of the computation times, measured in CPU ticks, is illustrated in Figure 7 below.

To have a better idea about the improvement, we performed some experiments to find if there was any overhead of chameleon signatures over traditional signatures. The overhead would comprise the computation of the chameleon hash and the computation of collisions on a new message. We used a traditional signature scheme, RSA 2048 bits, with message digest SHA256. We performed an experiment of computing the collision for a new message versus computing the signature of the SHA256 hash of the new message. We also compared with the elliptic curve signing scheme using the OpenSSL implementation of the curve secp256k1 defined in the Standards for Efficient Cryptography (SEC) (Certicom Research, http://www.secg.org/sec2-v2.pdf) and used in Bitcoin’s public-key cryptography. Finally, we included the advanced factorization method proposed by Shamir in [75] and implemented by Vishwas Patil (http://wwwusers.di.uniroma1.it/~patil/projects/cham/code.html) using OpenSSL.

In our approach, the DNS zone (or the delegated pattern RR) was signed only one time by the core DNS, using the chameleon hash computed by the fog DNS. Every time the zone required a change, the fog DNS only had to compute a collision using the previous chameleon hash so that the signature held. If a new fog DNS is introduced, it has to generate a new chameleon hash and have it signed by the core DNS. In the traditional approach, each change to the DNS requires the core DNS to perform one signature.

**Figure 8:**
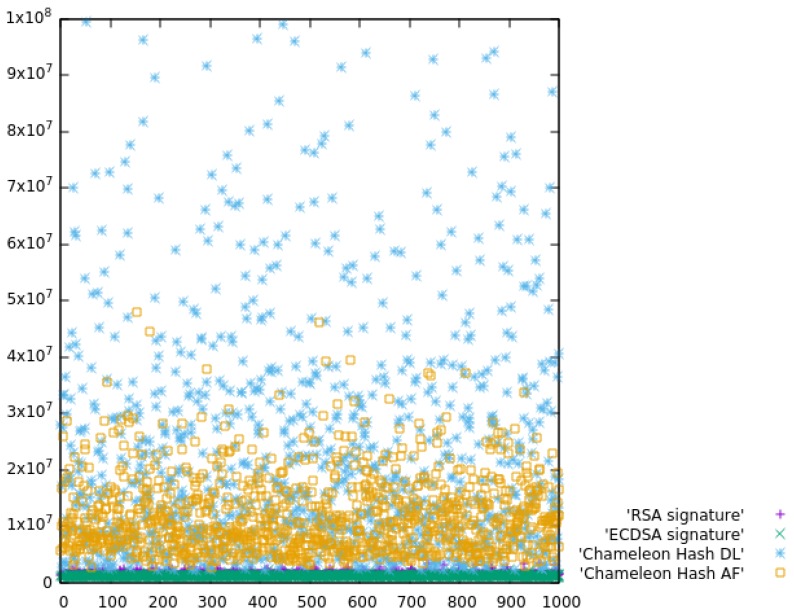
Signature computation time versus computing chameleon signature (y-axis in CPU ticks).

The experiment measured the performance of: (a) digitally signing a DNS RR with RSA, (b) with ECDSA, (c) computing a chameleon hash and finding a collision using discrete logarithms as explained in Section 5, and (d) using Shamir’s advanced factorization method. The times were measured in CPU ticks in an i9-8950H K@ 2.90 GHz. We repeated the experiment 1000 times. Figure 8 compares the ticks required for signing versus computing the chameleon hash. The y-axis is the time in CPU ticks, and the x-axis is the sample number. Each dot represents a computation, and the relatively large number of outliers is due to other activities performed by the operating system. We repeated the experiments a large number of times, but we were unable to avoid the outliers while finely measuring the time consumption. We used median values in the comparison. This comparison is meaningful when the domain name is soft delegated, which requires computing the chameleon hash and signing the pattern. The new process was slower than having the core DNS creating and signing a new DNS record. Concretely, RSA signing was 17-times faster (in median) than the proposed scheme, and seven-times faster if we used the advanced factorization proposal for chameleon hash of [75]. Of course, our process does scale, while having the core DNS performing the signatures will throttle the core DNS performance, and it does not scale.

This overhead is only introduced when a new fog DNS is created (new soft delegation). When microservice creation, re-instantiation, etc., do not require a new fog DNS, the advantage of our proposal is reflected in Table 10. Though at first sight, the left part implies more steps, they are far less complex than the steps in the right part. In fact, the numerical comparison in CPU ticks is shown in Figure 7. Since we are saving one signature time, every change to the DNS is secured faster with the proposed approach: 16-times faster using discrete logarithms and 28-times faster using Shamir advanced factorization. In IoT environments where the DNS will frequently change, this improvement is very significant, minimizing the computation required from both fog DNS and core DNS.

## 8. Conclusions

IoT presents several challenges: a notable increment of the background traffic in the core of the network due to signaling of subscribe/notify models, a lack of security support due to the limited nature of devices, and the overall difficulty in updating the security support of IoT devices. The adequacy of microservice architectures for IoT will alleviate the background signaling if deployed in the fog. Nevertheless, the proposed certificate pinning schemas do not offer the dynamicity required by microservices.

In this paper, we proposed a DNS-based dynamic authentication for a microservice architecture in IoT. We proposed using a soft delegation schema, with a DNSSEC forwarder at the fog. This fog DNS can perform frequent updates to the DNS to cope with the demand of microservice creation, migration, and re-instantiation. We proposed using a transferable, key exposure-free scheme of chameleon signatures, with a low computation cost for the fog forwarder and verifiers, which used DANE to offer the TLSA records, which can be used for establishing the TLS secured sessions for microservices to interact.

Regarding the dynamicity of changes, DNS addresses changes by means of the TTL of DNS resource records, so the TTL must be long enough to avoid frequent requests and short enough to ensure changes will be propagated promptly. In the case of the fog DNS, we envisage short TTLs, at the price of more frequent requests from clients, but ensuring changes will be rapidly known by the clients. The changes required by the orchestrator may require: moving a microservice to (1) a different node or (2) to new IPs within the same node. (1) will imply the fog DNS of the new node being soft delegated the domain name and computing the chameleon hash with the trapdoor function of the fog DNS of such a node. The TTL mechanism will be enough to ensure the previous fog DNS is not taking charge of the domain name any more. (2) will be addressed by the fog DNS computing a new collision and publishing the new record; this is the case when the creation of a new fog DNS is not required to support microservice creation, re-instantiation, etc., and the proposed mechanism achieves significative performance improvements.

Some TLS connections can have a longer life than established by DNS TTL. In the case of the instantiation of new copies of a highly-demanded microservice for load balancing, we were able to quickly disseminate the TLSAs records of the new instances. However, existing connections will persist and make it more difficult to balance the load with the fresh copies of the microservice. This has to be addressed with the support of a microservice infrastructure. Besides these future works, there remain some open problems, most notably the outdated support of PKI certificates in low-end devices. This makes it difficult to setup the initial connection securely prior to microservices’ deployment.

## Figures and Tables

**Table 1:** Example of a pattern record with placeholders.

Domain Name	TTL	Class	Type	Value
<T>[.L].xyz.com.	MAX_TTL	IN	<R>	rnd

**Table 2:** Example of pattern record with placeholders and usage. RR, Resource Record.

Domain Name	TTL	Class	Type	Usage
<T>[.L].xyz.com.	MAX_TTL	IN	<R>	Pattern scheme
<T>.xyz.com.	3600	IN	A	Allows to verify RRs of type Address (A) for domain xyz.com (like example.xyz.com). The TTL is limited to 3600
<T>.fog1.xyz.com.	1800	IN	A	Allows to verify RRs of type Address (A) for domain fog1.xyz.com (vending_z34_backup_service.fog1.xyz.com) it would not match A RR of the domain xyz.com
<T>.xyz.com.	720	IN	TLSA	Allows to verify TLSA records for the domain xyz.com

**Table 3:** Pattern record generation.

*S* serializes the pattern record: RR(i) = owner | type | class | TTL | RDATA length | RDATA, where RDATA=<T>.xyz.com|rnd. Resulting in msg = owner | type | class | TTL | RDATA length | <T>.xyz.com | rnd =RR(n) *S* chooses random *r* and *s*. *S* computes the pattern record hash ${\mathrm{hash}}_{\mathrm{SHA}256}\left(msg\right)=m$, and $e={\mathrm{hash}}_{\mathrm{SHA}256}\left(m,r\right)$ *S* computes the chameleon hash with the public key of the fog DNS (*R*): $\mathrm{CHAM}-{\mathrm{HASH}}_{R}\left(m,r,s\right)=\mathrm{chash}\left(m,r,s\right)=r-\left(y^{e}g^{s}\mathrm{mod}\;p\right)\mathrm{mod}\;q={\mathrm{RR}}_{\mathrm{PATTERN}}$ Finally, the RRSIG is computed by *S* as mandated by DNSSEC signature = sign(RRSIG_DATA | RR(1) | RR(2)... ) including *r* and *s*. The signature (in the example RSA-SHA256) computes the hash $\mathrm{shash}={\mathrm{hash}}_{\mathrm{SHA}256}{}_{(\mathrm{RRSIG}\_\mathrm{DATA}\;|\;r\;\;|\;s\;|\;}{\mathrm{RR}}_{\mathrm{PATTERN}})$. Then, it is cyphered with $SK_{S}$ to obtain the signature $\mathrm{sig}=\mathrm{sig}({}_{\mathrm{RRSIG}\_\mathrm{DATA}}|r|s|{\mathrm{RR}}_{\mathrm{PATTERN}})$ The signature, message, and random numbers are sent to fog DNS to be included in the domain zone data: (sig,r,s,msg). That record is formatted according to DNSSEC. The algorithm ID is 254 (PRIVATEOID), which allows a different verification mechanism. An example is given in Table 4.

**Table 4:** Example of a signed pattern record.

Domain Name	TTL	Class	Type	Value
<T> .xyz.com	3600	IN	A	32_bit_random_number (rnd)
<T> .xyz.com	3600	IN	RRSIG	**A 254** 3 3600 20170509183619 (20170409183619 32478 xyz.com. b64enc$\left(\mathrm{sig}({}_{\mathrm{RRSIG}\_\mathrm{DATA}}|r|s\right|{\mathrm{RR}}_{\mathrm{PATTERN}}),r,s)$)

**Table 5:** Creation of a DNS record by the DNS fog.

find a new random k∈(1,q−1) compute the collision for the chameleon hash ($\mathrm{CHAM}-{\mathrm{HASH}}_{R}\left(m,r,s\right)={\mathrm{RR}}_{\mathrm{PATTERN}}$ as in Equation (1): $r^{\prime }={\mathrm{RR}}_{\mathrm{PATTERN}}+\left(g^{k}\mathrm{mod}\;p\right)\mathrm{mod}\;q$. serialize the record: ${}_{\mathrm{RDATA}}=\;$163.117.141.197, and ${\mathrm{msg}}^{\prime }=\mathrm{owner}\;|\;\mathrm{type}\;|\;\mathrm{class}\;|\;\mathrm{TTL}\;|\;\mathrm{RDATA}\;\mathrm{length}\;|\;{}_{\mathrm{RDATA}}$. That will be the $m^{\prime }$ of Section 5. compute $e^{\prime }={\mathrm{SHA}}_{256}\left({\mathrm{msg}}^{\prime },r^{\prime }\right)$, and $s^{\prime }=k-e^{\prime }x\mathrm{mod}\;q$. this ensures that $\mathrm{shash}={\mathrm{hash}}_{\mathrm{SHA}256}({}_{\mathrm{RRSIG}\_\mathrm{DATA}}\;|\;r^{\prime }\;\;|\;s^{\prime }\;|\;{\mathrm{RR}}_{\mathrm{SRV}.\mathrm{XYZ}.\mathrm{COM}})={\mathrm{hash}}_{\mathrm{SHA}256}({}_{\mathrm{RRSIG}\_\mathrm{DAT}}\;|\;r\;\;|\;s\;|\;{\mathrm{RR}}_{\mathrm{PATTERN}})$. The new A record can be added to the zone together with $r^{\prime }$ and $s^{\prime }$. That record will be formatted according to DNSSEC using a private algorithm as previously mentioned. The following table illustrates an example of the DNSSEC records. Note the signature value is the same (the signature is not altered), but the new values of $r^{\prime }$, $s^{\prime }$ are included to make it transferable.				
**Domain Name**	**TTL**	**Class**	**Type**	**Value**
srv.xyz.com	3600	IN	A	163.117.141.197
srv.xyz.com	3600	IN	RRSIG	**A254** 3 3600 20170509183619 (20170409183619 32478 xyz.com. b64enc$\left(\mathrm{sig}({}_{\mathrm{RRSIG}\_\mathrm{DATA}}|r|s\right|{\mathrm{RR}}_{\mathrm{SRV}.\mathrm{XYZ}.\mathrm{COM}}),r^{\prime },s^{\prime })$)

**Table 6:** Initial information obtained by the fog DNS upon a resolver (*Q*) request.

*Q* queries type A records of domain name xyz.com, and within the answer, it gets the pattern and the new record:				
**Domain Name**	**TTL**	**Class**	**Type**	**Value**
<T>.xyz.com	3600	IN	A	32_bit_random_number
<T>.xyz.com	3600	IN	RRSIG	**A 254** 3 3600 2017050918361 (2017040918361 32478 xyz.com. b64enc(sig, *r*, *s*))
srv.xyz.com	3600	IN	A	163.117.141.197
srv.xyz.com	3600	IN	RRSIG	**A 254** 3 3600 2017050918361 (2017040918361 32478 xyz.com. b64enc(sig, $r^{\prime }$, $s^{\prime }$))

**Table 7:** Resolver (*Q*) verification of the DNS record.

According to [69], *Q* knows ZSK and verifies that: RRSIG RR and other records have the same owner, class, and type; the zone name in the RRSIG matches the record to verify; values of expiry, start, and TTL fields are consistent; verifies the signature algorithm to match with the DNSKEY, and the key is a ZSK; *Q* verifies the signature value of the pattern RRSIG record (retrieving *r*, *s* and computing msg, *e*, and the chameleon hash, applying the signature hash to get *chash*, verifying the signature $VRFY_{PK_{S}}\left(sig\right)=chash$), and checking that it is the same value as in the srv.xyz.com RRSIG record. *Q* verifies that ${\mathrm{RR}}_{\mathrm{PATTERN}}$ matches the ${\mathrm{RR}}_{\mathrm{SRV}.\mathrm{XYZ}.\mathrm{COM}}$ record checking the domain name, maximum TTL, and record type. *Q* computes ${}_{\mathrm{RDATA}}=$163.117.141.197, and ${\mathrm{msg}}^{\prime }=\mathrm{owner}\;|\;\mathrm{type}\;|\;\mathrm{class}\;|\;\mathrm{TTL}\;|\;\mathrm{RDATA}\;\mathrm{length}\;|\;{}_{\mathrm{RDATA}}$. *Q* retrieves $r^{\prime }$, $s^{\prime }$, and sig from the RRSIG record. *Q* computes ${\mathrm{hash}}_{\mathrm{SHA}256}\left(msg^{\prime },r^{\prime }\right)=e^{\prime }$ *Q* computes $r^{\prime }-\left(y^{e^{\prime }}g^{s^{\prime }}\right)\mathrm{mod}\;p)\mathrm{mod}\;q={\mathrm{RR}}_{\mathrm{PATTERN}}$ and $r-\left(y^{e}g^{s}\right)\mathrm{mod}\;p)\mathrm{mod}\;q={\mathrm{RR}}_{\mathrm{SRV}.\mathrm{XYZ}.\mathrm{COM}}$ and checks that ${\mathrm{RR}}_{\mathrm{SRV}.\mathrm{XYZ}.\mathrm{COM}}={\mathrm{RR}}_{\mathrm{PATTERN}}$; if they match, only the fog DNS private key $SK_{Fog}=x$ can compute the collision. **This verification proves that the record has been generated by the fog DNS authorized by the core DNS.**

**Table 8:** Fog DNS soft delegation.

Entity	Action	Complexity
Orchestrator	create fog DNS	-
Fog DNS	generate key pair; send public key (*y*) to core DNS	key creation
Core DNS	create and sign Key Tag (KTR)	$sig_{KSK}\left(KTR\right)$
Core DNS	create pattern, compute CHAM-HASH	chameleon hash
Core DNS	sign RRSIG	$sig_{ZSK}\left(\mathrm{RRSIGDATA}.\right)$

**Table 9:** Comparison of the proposal: new microservice.

Entity	Action	Signature
Orchestrator	Send (name and address) to the fog DNS	-
Fog DNS	compute the collision, and create RR and RRSIG reusing the original signature	-

**Table 10:** Comparison between signing and finding a collision.

Finding a Collision in the Chameleon Hash	Signing a New Message
Given new M’ and $H={\mathrm{CHAM}-\mathrm{HASH}}_{R}\left(M,r,s\right)$	
find random k∈(1,q−1) compute $A=g^{k}\mathrm{mod}\;p$ compute $r^{\prime }=A+H\mathrm{mod}\;q$. compute $e^{\prime }={\mathrm{SHA}}_{256}\left(M^{\prime },r^{\prime }\right)$ compute $s^{\prime }=k-xe^{\prime }\mathrm{mod}\;q$.	Given new M’ $e^{\prime }={\mathrm{SHA}}_{256}\left(M^{\prime }\right)$ $sig=SIG_{SK_{S}}\left(e^{\prime }\right)=e^{\prime d}\mathrm{mod}\;n$.
